# The Evolution of Your Success Lies at the Centre of Your Co-Authorship Network

**DOI:** 10.1371/journal.pone.0114302

**Published:** 2015-03-11

**Authors:** Sandra Servia-Rodríguez, Anastasios Noulas, Cecilia Mascolo, Ana Fernández-Vilas, Rebeca P. Díaz-Redondo

**Affiliations:** 1 Computer Laboratory, University of Cambridge, Cambridge, United Kingdom; 2 I&C Laboratory, AtlantTIC Research Center, University of Vigo, Vigo, Spain; Technical University Darmstadt, GERMANY

## Abstract

Collaboration among scholars and institutions is progressively becoming essential to the success of research grant procurement and to allow the emergence and evolution of scientific disciplines. Our work focuses on analysing if the volume of collaborations of one author together with the relevance of his collaborators is somewhat related to his research performance over time. In order to prove this relation we collected the temporal distributions of scholars’ publications and citations from the Google Scholar platform and the co-authorship network (of Computer Scientists) underlying the well-known DBLP bibliographic database. By the application of time series clustering, social network analysis and non-parametric statistics, we observe that scholars with similar publications (citations) patterns also tend to have a similar centrality in the co-authorship network. To our knowledge, this is the first work that considers success evolution with respect to co-authorship.

## Introduction

*Success*, according to the *Merriam Webster* dictionary, is “getting or achieving wealth, respect, or fame”. But, how can we measure *wealth, respect* or *fame* and, ultimately, *success*? These concepts are subjective and, as Barabási claimed, success is a collective phenomenon in the sense that a person (or other entity) is successful because others around him believe that he is (http://www.northeastern.edu/news/2013/06/scienceofsuccess/). Despite this, some objective measures have been proposed for quantifying success, measures that depend on the domain (context) in which success is assessed. For instance, if we think about success in the marketing domain, a campaign will be successful if it gets to increase the profit. On the contrary, in social media, we could quantify the success of a video in YouTube by means of the number of views, the success of a tweet by the number of retweets received or the success of a Twitter user by the number of users who follow him (his followers).

In this paper we study academic researchers success: academic success can be tracked through the monitoring of the publication record of the academics in conferences and journals over the years. Many tools exist to gather data about published articles and citations and we will rely on these for our study. More specifically in this research we will focus on (i) the dissemination of results and (ii) the attention that these results receive in the research community as objective signs of scholars’ success. That is, we will focus on the publications (be conference papers, journals, books or patents) that authors get and the citations that these publications receive. We will also focus on scholars’ collaborations, and more specifically, on analysing if the volume of collaborations of one author, together with the relevance of his collaborators, is somewhat related to his research performance over time (temporal success). Therefore, the two main innovative factors of this work are *the temporal evolution* and *the relationship of one author’s success to the success of his collaborators*:

- *Temporal evolution*: an important factor when assessing the success of an author should be the variability in the diffusion of their achievements over time. We study the temporal variation of an author’s publications and citations record: the number is not the only important measure, but the pace of publication activity and the impact of this activity are also very relevant.
- *Collaboration*: the collaboration among researchers [1, 2] or their institutions [3–5] drives researchers success. As an outstanding example, the recent study of Bellotti [5] indicates that, in order to be successfully funded, the interaction over the years with different research groups counts more than working in a large university. Although this study is limited to research projects in the physics discipline funded by the Italian Ministry of University and Research, a look at the collaboration clusters winning research grant calls seems to confirm this informally too.

In order to prove the relation between the volume of publications/citations of one author over time and the collaboration network of co-authors of his papers, we collected the temporal distributions of scholars’ publications and citations from the Google Scholar platform (http://scholar.google.com/) and the co-authorship network (of Computer Scientists) underlying the well-known DBLP bibliographic database (http://www.informatik.uni-trier.de/∼ley/db/). Although Google Scholar contains the temporal distributions of all the publications and citations of each author (at least the ones from venues indexed by Google Scholar), the fact that not all the authors have a profile in this platform hampers us from having the complete view of the real collaboration network (henceforth, co-authorship network). On the contrary, the DBLP bibliographic database, despite only including Computer Science researches, provides a complete view of the co-authorship network, both in terms of nodes (scholars) and edges. However, this bibliographic database does not include citations data and therefore cannot be used for analysing the problem by itself alone. As a solution, we combine the aforementioned datasets: we apply time series clustering on the citations data of Google Scholar obtaining authors clusters; we then consider a collaboration network extracted from DBLP and apply social network analysis on it. We observe that the (median) centrality of those scholars with similar publications (citations) patterns (i.e. in the same cluster) is different from the (median) centralities in the rest of clusters. These findings open the door to potentially new publishing strategies as well as prediction of success for young scholars, potential recruits as well as journal editors.

The main novelty of our approach is to *consider the whole scholars’ timeline as sign of their research activity*. Although both citation and publication counts have been widely used as indicators of scholars’ impact [6–8], most of the existing metrics only consider one time window which may be difficult to select as it depends on several cross-cutting factors [9, 10]. Given the importance of time of publications/citations in determining success, the complete timeline of the scholars may give a more complete view. Moreover, our work establishes scholars’ collaboration according to their relative importance within the co-authorship network (centrality) as the most formal sign of academic teamwork. In order to establish the dependence among the longitudinal data (time series of citations and publications) by obtaining some derivable variable from the series, we propose retaining all the information of the temporal research activity and exploring temporal patterns for scholars by clustering their timelines. Then, after studying the *degree, closeness, betweenness* and *eigenvector* centrality measures considering the collaboration network for the clustered authors and observing that they are far from being normally distributed, we use non-parametric statistics (Kruskal-Wallis statistical test [11]) in order to check whether there are differences, in terms of authors’ centrality, among the different clusters. That is, whether the median centrality of the authors classified in the same cluster is different from the median centralities in the rest of clusters.

The main findings of our study are:

- The volume of publications/citations over time of one Computer Scientist who started his career between 1979 and 2009 is related to the volume and relevance of his collaborators. That is, to his centrality in the co-authorship network.
- This relation holds for most of the period considered, which means that collaboration affects (and is affected by) research performance over time. What is likely is that, the more collaborations one author has, the higher his number of publications will be and the more attention they will receive.

## Materials and Methods

### Datasets collection

We now describe the two datasets we have worked with and the reasoning behind this choice.

- The Google Scholar platform allows academics to create profile pages that contain, in addition to their affiliation and areas of interest, their history of publications and citation counts over time. A profile page in this platform allows other academics to see, at a glance, the author’s publications without having to search in the traditional Google Scholar page. Also, and more importantly, instead of only showing the total number of citations of a paper (author), it displays the citations distribution of the paper (author) over time. Google Scholar also allows researchers to link to collaborators, however this function is not very popular and therefore it is almost impossible to gather collaboration network data from Scholar.
- To complement Google Scholar and be able to obtain collaboration network information, we used the *DBLP Computer Science Bibliography*, a tool developed by researches from the *University of Trier*: with this, we trace co-authorship in the work of Computer Science researchers. Although considering only Computer Science researchers limits the analysis, this gives us a complete network of co-authors to work with.

We now describe how we obtained the data.

### Google Scholar

Every author in Google Scholar has his own identifier, but there is no way to know all of the identifiers of Google Scholar authors. So, one indirect way for getting the given identifiers (or at least most of them) consists of crawling Google Scholar through the connections among the co-authors that authors have previously included in their profiles. But, as aforementioned, the number of authors that have explicitly indicated their co-authors is very small. However, many of them do have indicated their areas of research. As Google Scholar allows to query authors interested in a given area, if we knew all the areas of interest in Google Scholar, we could get all authors’ identifiers (at least the ones that have indicated their areas of interest). Again, as authors can freely indicate their areas of expertise (there is not a predefined group of them) there is not direct way to know all the areas of interest in Google Scholar. We have devised an algorithm that, starting with an author’s profile (acting as seed), retrieves his areas of interest and all the authors that have indicated the same areas as him. It then randomly selects one of these authors and repeats this procedure, retrieving the areas and the authors that have not been crawled yet. The algorithm finishes when there are not more authors to select.

Our dataset contains information about 192,930 authors with profile in Google Scholar and, at least, one publication indexed by Google Scholar and 9,030,060 papers. Table 1 summarises (i) authors’ and (ii) papers’ attributes contained in this dataset (previously retrieved from the Google Scholar platform).

**Table 1:** Google Scholar profiles dataset.

**Author’s information**	**Paper’s information**
name	title
affiliation	authors (name, not links to authors’ ids in Scholar)
domain	publication type (conference, journal, patent)
citations and publications time series	publication name
total number of citations (after 2008 and all)	publication date
*h* index (after 2008 and all)	publisher
*i*_10_ index (after 2008 and all)	citations time series
areas of interest	total number of citations

### DBLP

The DBLP Computer Science Bibliography is a tool developed by researches from the University of Trier to trace the work of Computer Science researchers. The whole DBLP content is available, in form of XML file, in *http://dblp.uni-trier.de/xml*, whose structure is described in *http://dblp.uni-trier.de/xml/dblp.dtd*. An updated version of the XML file is released daily. As of 2013, the dataset includes 1,227,149 authors and 9,822,354 co-authorship relations.

The information in this dataset is presented in a publication-centred perspective. Different publications are considered, following a *Bibtex* style, as: *article, inproceedings, proceedings, book, incollection, phthesis, mastherthesis* or *www*. Data associated to each publication are shown in Table 2. The relevant information for our purposes is authors’ names and publications, which will allow us to obtain the co-authorship network. That is, the network in which nodes are the computer science authors and a link between them exists if they have co-authored, at least, one publication.

**Table 2:** DBLP dataset: publication’s information.

title	publication date	year	number	school	chapter
authors’ (editors’) name	publication type	journal	month	publisher	series
authors’ (editors’) address	pages	volume	url	note	isbn

The aforementioned datasets (Google Scholar and DBLP) have different levels of coverage: while Google Scholar contains information about researchers in whatever area of expertise and who have created a profile in this platform, DBLP contains information about all the researchers in the Computer Science domain (or at least about those that have published, at least once, in a conference/journal indexed by DBLP). The two platforms associate users to different identifiers which means that we have to match authors in both platforms. To this aim, we applied a conservative solution: the lexicographic comparison of authors’ names in both datasets, obtaining that the number of authors with profile in both datasets is 57416, which represents around 30% of our entire Google Scholar dataset.

## Analysis Techniques

As mentioned, one of the main contributions of this work is the ability to track the evolution of authors’ success through the use of the temporal data contained in our datasets. We analyze the data longitudinally without resorting to aggregates or univariate summaries (e.g. averages or time slopes). Our approach can be briefly described as follows: we first apply hierarchical clustering to the Google Scholar longitudinal data containing citations and publications, operation that divides our authors into groups with similar patterns of publications (citations). We then consider the DLBP collaboration network and apply social network analysis to determine author centrality. Finally we study the relation between the author centralities and the clusters. In the next sections we detail the various steps we just described.

### Time Series Analysis

We start by exploring the longitudinal dataset to discover groups of authors that share similar publication/citation dynamics as a mean to identify different patterns of success evolution in the pool of authors. For that, among unsupervised techniques for exploratory data analysis, clustering is a strong candidate for finding strongly related data points. An ample variety of algorithms have been proposed for clustering purposes (see [12] for a review). Given the scarce knowledge about temporal patterns in scholars’ activity to date and considering its conceptual simplicity and good scaling with a large number of points, a hierarchical clustering algorithm (*The Unweighted Pair Group Method with Arithmetic Mean (UPGMA)* [13]) seems appropriate as it constructs a rooted tree (dendrogram) for inspection. This dendrogram reflects the structure present in a pairwise similarity matrix (or a dissimilarity matrix) by accomplishing iterative steps as follows:

1. Place each data point into its own singleton group.
2. Merge the two closest groups.
3. Update distances *D* between the new cluster (group) and each of the old clusters (see below).
4. Repeat 2 and 3 until all the data are merged into a single cluster.

Given a distance measure *d* between points UPGMA obtains the intergroup similarity between the clusters *C* and *H* as:

$$D\left(C,H\right)=\frac{1}{N_{C}N_{H}}\sum_{i\in C}\sum_{j\in H}d_{i,j}$$ (1)

where *N_C_*(*N_H_*) is the size of the cluster *C*(*H*) and *d_i,j_* is the distance between the data points *i* and *j*.

For the case of UPGMA over time series, the pairwise similarity matrix is composed of similarity measures between every two time series (authors’ time series of publications or citations as corresponds). Our methodology constructs this matrix by applying the well-known *Dynamic Time Warping (DTW)* method [14], which finds an optimal alignment between two time-dependent sequences *S*_1_ and *S*_2_ by warping the time dimension in *S*_1_ that minimises the difference between the two series so that time series are not need to be of equal length. Although DTW was initially applied to word’s recognition continuous human speech [15, 16], its use has been extended to other domains. In our experiment, we alleviate the problem of alignment by classifying authors in advance by their first publications’s year, so that only time series of equal length are considered.

DTW works as follows. Let us suppose that we have two time series *S*_1_ = {*s*_1_1__, *s*_1_2__, …, *s*_1_*n*__} and *S*_2_ = {*s*_2_1__, *s*_2_2__, …, *s*_2_*m*__} that represent the temporal distribution of publications (citations) of author 1 and author 2 respectively. If *i* = 1, 2, …*n* and *j* = 1, 2, …, *m* are indices into *S*_1_ and *S*_2_ and *W* = *w*_1_, *w*_2_, …, *w_K_*, is an optimal mapping between the two sequences given as,

$$\begin{array}{c} w_{k}=\left(w_{k_{x}},w_{k_{y}}\right)\\ k=1,2,...,K\\ w_{k_{x}}\in \left\{1,2,...,n\right\}\\ w_{k_{y}}\in \left\{1,2,...,m\right\} \end{array}$$ (2)

we can compute the dissimilarity or distance between the two sequences as,

$$d\left(S_{1},S_{2}\right)=DTW\left(S_{1},S_{2}\right)=\underset{W}{\text{min}}\left\{\sum_{k=1}^{K}\delta \left(w_{k}\right)\right\}$$ (3)

where δ(*w_k_*) is a non-negative function to compute dissimilarity between individual elements of *S*_1_ and *S*_2_.

As most clustering algorithms, the key issue in UPGMA is fixing the number of resulting clusters. Being UPGMA a hierarchical method, this turns into the problem of identifying the relevant branches of the cluster tree (dendrogram pruning). In order to not have to decide for each individual branch, the most widely used technique is based on fixing the height of the dendrogram so that each contiguous branch below that height is considered a separate cluster. Unfortunately, selecting this height is not a trivial task and, alternatively, *Dynamic Tree Cut* (DTC) [17] proposes to prune the dendrogram by taking its shape into consideration. DTC iteratively applies decomposition and combination of clusters until their number becomes stable. After obtaining a few large clusters by the fixed height branch cut method, the joining heights of each cluster are analysed for a sub-cluster structure. Clusters with this sub-cluster structure are recursively split and, with the aim of avoiding over-splitting, very small clusters are joined to their neighbouring major clusters.

### Social Network Analysis

Once we have discovered the structure of the temporal dataset, the next step is to establish if this structure is related to scholars’ centrality in the co-authorship network. For that, we take advantage of the DBLP dataset to obtain the current network of co-authorship. We represent this network as an undirected graph *G* = (*V, E*), where *V* denotes the set of scholars (authors) in the dataset, and an edge *e* ∊ *E* between two scholars *u, v ∊ V* exists if they have co-authored, at least, one publication. We will then be able to determine if the position of the author in the given network has relation with his citations (publications) behaviour in Google Scholar. That is, if the fact that two authors are included in the same cluster implies that they have similar centrality (we have tried various measures of centrality) in the network. This will indicate if researchers’ collaboration activity is dependent on the temporal distribution of the publications or the citations that they receive. With this aim, we applied social network analysis over the co-authorship network extracted from DBLP.

The next step consists in determing the centrality of each node within the network. Several measures of centrality have been proposed to date. The most fundamental and popular definitions of centrality were proposed by Freeman [18]. By this definition, the centrality is measured based on *degree, closeness* and *betweenness*. These measures, together with the *eigenvector* centrality, are detailed below.

- **Degree centrality**: The degree of a node *n_i_* in a graph is the number of direct connections that a node has. It is the simplest and easiest way of measuring its centrality, being a local measure of a node’s importance. The degree centrality of a node reflects the popularity and relational activity of the node.
  $$C_{D}\left(n_{i}\right)=\sum_{i=1}^{N}a\left(n_{i},n_{k}\right)$$ (4)
  where *N* is the number of nodes in the network and *a*(*n_i_, n_k_*) is a distance function. *a*(*n_i_, n_k_*) = 1, if and only if node *n_i_* and node *n_k_* are connected. *a*(*n_i_, n_k_*) = 0 otherwise.
- **Betweenness centrality**: The betweenness centrality of a node *n_i_* is defined to be the fraction of shortest paths between pairs of nodes in a network that pass through *n_i_*. It represents the node’s capability to influence or control interaction between nodes that it links.
  $$C_{B}\left(n_{i}\right)=\sum_{n_{j}<n_{k}}^{N}g_{n_{j}n_{k}}\left(n_{i}\right)/g_{n_{j}n_{k}},n_{i}\neq n_{j}\neq n_{k}$$ (5)
  where *g_n_j_n_k__* (*n_i_*) is the number of shortest paths connecting *n_j_* and *n_k_* passing through *n_i_* and *g_n_j_n_k__* is the total number of shortest paths connecting them.
- **Closeness centrality**: The closeness centrality of a node measures how many steps are required to reach a given node from every other node. It indicates the node’s availability, safety and security depending on the application context considered.
  $$C_{C}\left(n_{i}\right)={\left[\sum_{i=1}^{N}d\left(n_{i},n_{k}\right)\right]}^{-1}$$ (6)
  where *N* is the number of nodes in the network and *d*(*n_i_*, *n_k_*) is the number of hops in the shortest path between *n_i_* and *n_j_*.
- **Eigenvector centrality**: The eigenvector centrality is a measure of the importance of a node in a network and it is based on the idea that a node is more central if it is in relation with nodes that are themselves central.
  $$C_{E}\left(n_{i}\right)=\frac{1}{\lambda }\sum_{k=1}^{N}a\left(n_{i},n_{k}\right)C_{E}\left(n_{k}\right)$$ (7)
  where λ is a constant, *N* is the number of nodes in the network and *a*(*n_i_, n_k_*) is a distance function. *a*(*n_i_, n_k_*) = 1, if and only if node *n_i_* and node *n_k_* are connected. *a*(*n_i_, n_k_*) = 0 otherwise.

The next section shows the results of our analysis.

## Results

Our methodology for analysing the data obtained from both Google Scholar and DBLP processes each dataset separately. Then, the results are analyzed through statistical tests to determine if there exists any relation between them. In the case of Google Scholar, we also process separately publications and citations, as they are different indicators of authors’ success: whereas a publication is reviewed by a group of previously selected experts in the domain (reviewers), any author, in or out of the domain, can cite a previously published publication. On the other hand, authors have started to publish (receive citations) in different years and their publications and citations are conditioned by the state of the research environment in every moment of their trajectory. The number of existing conferences and journals or the accessibility to bibliographic resources are only some of the factors that condition authors’ publications and citations in a given period. To avoid the impact of this aspect on the results, we decided to split authors in groups depending on the year in which they started to publish (in the case of publications) or to receive the first citation (in the case of citations). In the case of DBLP, as the co-authors network is the result of collaborations between authors through the years, we work with the whole network.

**Fig 1:**
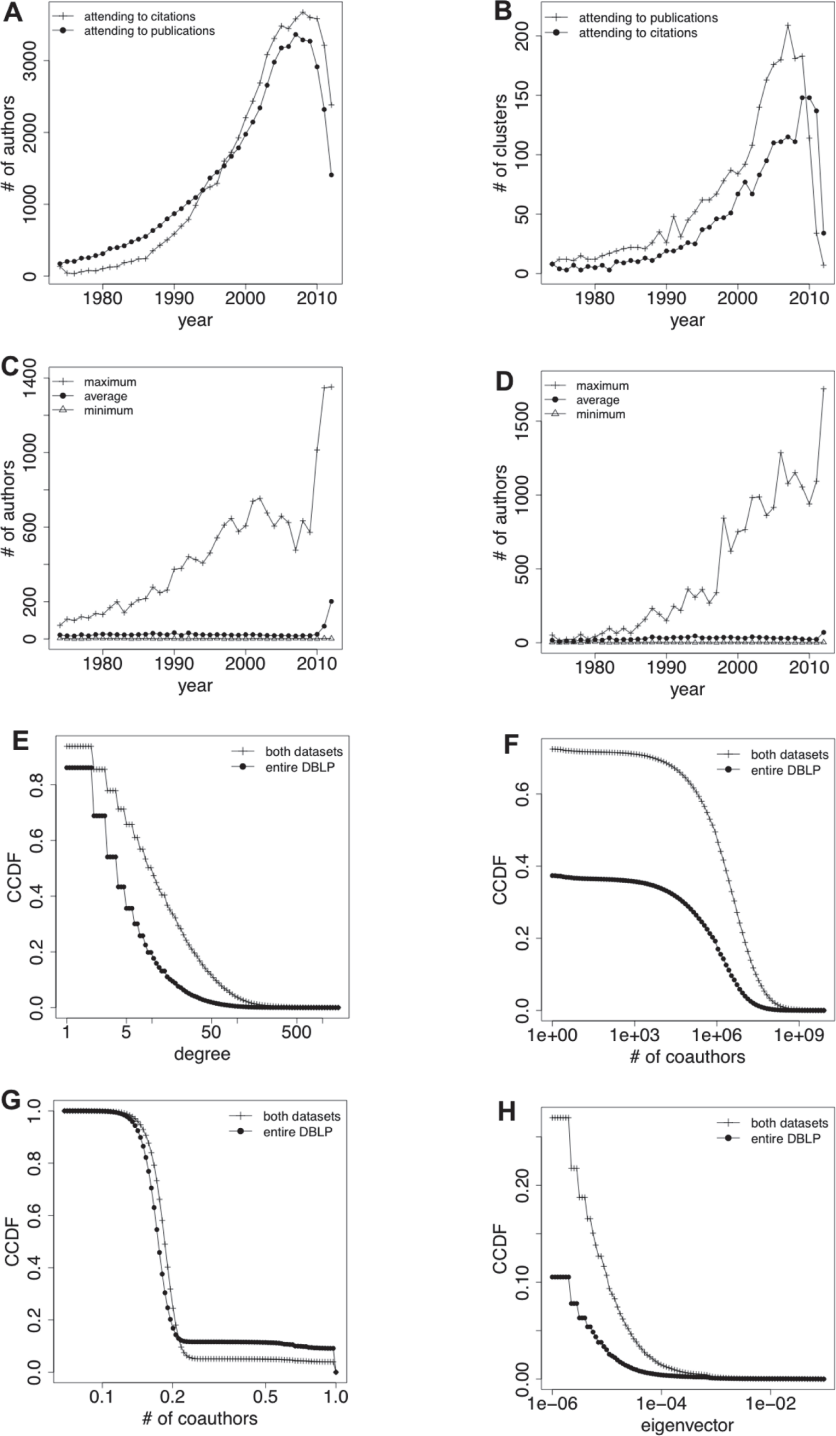
Time series clustering in Google Scholar and Centrality measures in DBLP. (a) Number of authors attending to the year of their first publication (citation). (b) Number of clusters of authors per year. (c) Number of authors per cluster per year (attending to the temporal evolution of their publications). (d) Number of authors per cluster per year (attending to the temporal evolution of their citations). (e) Degree centrality in the co-authorship network (for authors simultaneously in *both datasets* and for authors in the *entire DBLP*). (f) Betweenness centrality in the co-authorship network (for authors simultaneously in *both datasets* and for authors in the *entire DBLP*). (g) Closeness centrality in the co-authorship network (for authors simultaneously in *both datasets* and for authors in the *entire DBLP*). (h) Eigenvector centrality in the co-authorship network (for authors simultaneously in *both datasets* and for authors in the *entire DBLP*).

With respect to the distribution of authors by year of their first publication (citation), Fig. 1A represents the number of authors classified by (i) the year in which they achieved their first publication and (ii) the year in which any of their publications received its first citation. Both the Computer Science discipline and the Google Scholar platform are relatively recent in comparison to other scientific disciplines and other scientific databases, which could explain why the curves of the number of authors per year are increasing.

### Grouping authors

As aforementioned, we applied hierarchical clustering over the time series of authors’ publications (citations) in order to extract groups of authors with similar publication (citation) patterns. Fig. 1 contains information about the resulting clusters: number of resulting clusters (Fig. 1B) and number of authors per cluster in the case of publications (Fig. 1C) and citations (Fig. 1D).

Focusing on Fig. 1B, we see that the number of resulting clusters increases with the year of the first publication (citation) and, therefore, with the number of time series (authors) to be clustered, at least until the late 2000s. That is, the more authors to cluster, the more different patterns emerge. Although this tendency is notable both in publications and citations, the increase is higher in the case of publications, highlighting the variability of the publication records. With respect to the number of authors (time series) per cluster, we appreciate that the resulting clusters have, in average and independently of the year, equal number of elements (authors). On the contrary, the curve of the maximum number of elements in a cluster is increasing with the number of authors in a cluster until mid-2000s. When there are more authors who started to publish (receive citations) in the same year, (i) the diversity in terms of publication/citation patterns is higher and (ii), although in average the number of authors with similar patterns (classified in the same cluster) keeps constant with respect previous years, there are some big groups of authors (clusters) that share a similar pattern of publications (citations).

However, and as aforementioned, there are certain periods in mid-2000s in which, although (i) the number of authors who started their activity around these years and (ii) the diversity in terms of citation/publication patterns are higher than in previous years, there are less authors that share similar patterns (less authors per cluster). A feasible explanation for this could be that, as these authors are relatively new to the research area, they are in a “transitory” period of their careers, which could cause the high diversity of behaviour among them. Finally, Fig. 1 also shows that recent scholars, those who start to publish/being cited between 2010 to 2012 and therefore have had less than 3 years to develop their research activity, are, in general, authors with very few publications/citations which makes that almost all of them present the same publication/citation pattern.

### Obtaining authors’ centrality

The CCDF (Complementary Cumulative Distribution Function) curves corresponding to the centrality measures previously described are included in Fig. 1. For each measure, we provide two curves: the one that represents the distribution of the given centrality for the authors in the whole DBLP dataset and the other that limits the curve to only those authors that simultaneously appear in DBLP and in Google Scholar dataset (those subject of our study).

Specifically, Fig. 1E shows the distribution of degree centrality among authors, that is, the number of authors with whom they have collaborated at least once. Although the tendency of the curve is similar in the case of all computer science authors or only computer science authors with profile in Google Scholar, the degree is slightly higher in the second case. Looking at the CCDF for the whole dataset, we see that around the 40% of the authors have a degree centrality value higher than 5, whereas when only authors in our Google Scholar dataset are considered this percentage increases to the 70%. This means that authors who own a profile in Google Scholar are more prone to co-author papers with different authors than the rest of computer science authors. In the case of eigenvector centrality (Fig. 1H) most of the nodes (authors) in the network have an eigenvector centrality equal to zero. That is, around the 11% of the authors in the whole dataset have a positive eigenvector centrality, whereas this percentage increases to the 27% when only authors in Google Scholar are taken into account. Talking in terms of research collaborations, this means that scholars are prone to collaborate with both, central and non-central authors, which means that collaborations are quite distributed across the network instead of being topologically focused on only one or a few areas in the network.

In the case of betweenness centrality (Fig. 1F), around the 30% of authors that are simultaneously in both datasets (Google Scholar and DBLP) have no shortest path through them, whereas this percentage increases up to almost 60% when the entire DBLP dataset is taken into account. This suggests that authors who own a profile in Google Scholar are more prone to serve as links between scholars (co-authorise papers with well-connected authors) than the rest of computer science authors. With respect to authors with positive values of betweenness, there is no difference between the distribution of this variable among the total authors in computer science (in the whole DBLP dataset) with respect to the distribution when only Computer Scientists with a profile in Google Scholar are considered. Finally, in the case of closeness centrality (Fig. 1G) the distribution of closeness centrality of authors in Google Scholar is similar to the one of all computer science authors. Specifically, the total of computer science scholars have a value of closeness higher than 0.12 and only the 10% of them have a value of closeness equal to 1, being this percentage reduced until the 5% in the case of authors with profile in Google Scholar. Talking in terms of co-authorship relations, this means that only a reduced percentage of scholars are at a reachable distance, in terms of co-authorship links, of the rest of computer science authors. But, the immense majority of authors is far from having collaborated with the rest of nodes in the network.

### Relating academic success and centrality: the Kruskal-Wallis test

The last step of our analysis deals with checking the existence of relation among the authors included in the same cluster and their centrality in the co-authorship network. To this aim, the common approach in the literature is the one-way analysis of variance [19] (one-way ANOVA), a statistical technique used to compare the means of two or more samples (being the samples, in our case, the authors classified into each cluster). Specifically, ANOVA tests the null hypothesis that samples in two or more groups are drawn from populations with the same mean values. In our scenario, this implies testing if the authors classified in the previously calculated clusters (*response* or dependent variable) are drawn from authors with the same mean values of centrality in the co-authorship network (*factor* or independent variable). But the reliability of one-way ANOVA results is conditioned, among others assumptions, by normality of the response variables, and the application of the Shapiro-Wilk statistical test [20] revealed that centrality distribution of authors within a cluster is far for being normally distributed. Under these circumstances, we opted for a non-parametric alternative to the one-way ANOVA, the Kruskal-Wallis test [11]. Contrary to one-way ANOVA, which worked with mean values, Kruskal-Wallis tests the null hypothesis that samples in two or more groups are drawn from populations with the same median values. Thus, we applied Kruskal-Wallis test to determine if the centralities of the authors with the same publication (citation) pattern had the same median value and if this value was different from the median value of authors with different publication (citation) patterns. That is, if authors with similar centrality in the network have similar publication (citation) patterns and these patterns are different from the ones from authors with different centrality values.

The Kruskal-Wallis statistical results considering *degree, betweenness, closeness* and *eigenvector* centrality measures for the different years are shown in Tables 3 and 4 for publications and citations respectively. Taking the standard *α* = 0.05 as the significance level for all the Kruskall-Wallis tests conducted, results revealed that the *p*-value is lower than the significance value, and therefore the null hypothesis can be rejected for the most relevant cases, those which includes authors whose starting publication date was between 1979 and 2009 (Table 3), for all the centrality with the exception of the closeness centrality during some years between 1979 and 1987. These results are highly conclusive since, for authors who started to publish between 2010 and 2012 (classified into two or more clusters attending to their publications rate), the length of their time series (2 or 3 points) is not enough for obtaining accurate values of similarity and, consequently, for a suitable (clustering) classification. A similar situation can be appreciated in authors that started to publish before 1978. In this period, there exists a huge diversity, both in terms of measures and years, in which the null hypothesis cannot be rejected. In any case, and due to the peculiarities of the Google Scholar dataset (that counts publications since 1974) and that Computer Science is a relatively recent discipline, the number of authors that started to publish before 1980 is quite limited with respect to the number of computer scientists that started their activity later and these results should not be considered relevant. Again, clustering for this period may not be considered reliable enough. With all of this, the conclusions so far are that (i) in around the 80% of the considered period authors’ centrality is highly related with the distribution of publications over time and (ii) the periods in which this relation is not determined correspond to periods in which there are very few authors or periods of temporal series of publications extremely short.

**Table 3:** Kruskal-Wallis statistics results for the publications case.

		*Degree*		*Betweenness*		*Closeness*		*Eigenvector*	
year	df	*χ* ^2^	*p*	*χ* ^2^	*p*	*χ* ^2^	*p*	*χ* ^2^	*p*
**1974**	7	10.0	0.187	10.6	0.155	13.0	0.071	16.1	0.024
**1975**	11	15.0	0.183	18.1	0.079	7.3	0.778	13.4	0.268
**1976**	12	43.4	1.9e-05	39.3	9.2e-05	21.2	0.048	34.6	0.001
**1977**	10	22.6	0.012	24.3	0.007	16.7	0.081	17.0	0.073
**1978**	14	24.6	0.039	19.5	0.148	14.1	0.444	28.7	0.011
**1979**	11	32.2	0.001	28.5	0.003	21.4	0.029	20.3	0.041
**1980**	11	55.1	7.4e-08	46.1	3.1e-06	21.6	0.028	40.2	3.2e-05
**1981**	14	35.5	0.001	29.8	0.008	20.4	0.117	34.1	0.002
**1982**	16	54.0	5.3e-06	55.5	2.9e-06	29.0	0.024	50.5	1.9e-05
**1983**	18	51.8	4.0e-05	57.5	5.2e-06	34.1	0.012	45.0	4.1e-04
**1984**	21	61.5	7.6e-06	53.5	1.2e-04	31.7	0.063	43.5	0.003
**1985**	21	81.2	5.2e-09	79.0	1.2e-08	41.4	0.005	43.1	0.003
**1986**	21	97.4	8.2e-12	105.3	3.3e-13	31.5	0.066	48.1	0.001
**1987**	20	97.3	3.9e-12	94.8	1.1e-11	29.9	0.071	51.7	1.3e-04
**1988**	26	144.3	2.3e-18	138.7	2.3e-17	62.0	9.1e-05	89.3	7.1e-09
**1989**	34	136.4	3.2e-14	136.6	3.0e-14	93.9	1.6e-07	102.2	9.5e-09
**1990**	25	178.0	5.0e-25	173.9	2.9e-24	75.6	5.5e-07	119.4	2.9e-14
**1991**	47	162.0	1.5e-14	149.5	1.3e-12	90.7	1.4e-04	89.7	1.8e-04
**1992**	31	181.8	3.0e-23	178.6	1.0e-22	70.4	6.8e-05	87.5	2.7e-07
**1993**	44	203.8	2.0e-22	211.7	8.5e-24	107.6	3.0e-07	126.0	7.8e-10
**1994**	51	279.4	3.0e-33	298.4	1.1e-36	112.6	1.5e-06	151.2	7.4e-12
**1995**	61	277.1	2.6e-29	269.6	5.2e-28	124.9	2.7e-06	169.8	3.3e-12
**1996**	61	233.2	6.0e-22	241.8	2.3e-23	134.6	1.8e-07	147.0	4.6e-09
**1997**	66	331.4	5.4e-37	328.4	1.8e-36	123.4	2.4e-05	170.7	3.3e-11
**1998**	78	315.6	2.5e-30	302.5	3.5e-28	176.2	1.5e-09	195.0	5.3e-12
**1999**	87	323.4	6.6e-29	324.6	4.2e-29	158.1	4.9e-06	178.9	2.5e-08
**2000**	84	339.0	2.4e-32	310.6	1.0e-27	150.8	1.1e-05	182.9	2.6e-09
**2001**	91	401.6	1.3e-40	382.3	2.3e-37	181.7	5.3e-08	176.6	2.0e-07
**2002**	107	390.4	7.1e-34	375.4	1.7e-31	204.6	4.3e-08	218.0	1.4e-09
**2003**	140	506.2	6.5e-43	452.9	1.2e-34	247.3	5.9e-08	239.1	3.7e-07
**2004**	162	498.2	6.9e-36	429.1	4.9e-26	284.7	8.8e-09	239.3	7.5e-05
**2005**	175	504.9	9.4e-34	407.7	1.3e-20	263.2	1.8e-05	222.9	0.008
**2006**	179	508.3	2.6e-33	445.3	1.1e-24	226.6	0.009	244.5	0.001
**2007**	208	512.7	1.9e-27	436.8	2.3e-18	258.3	0.010	256.2	0.013
**2008**	180	462.2	1.0e-26	380.3	2.0e-16	227.3	0.010	216.2	0.034
**2009**	183	433.6	2.2e-22	344.2	5.8e-12	228.7	0.012	218.1	0.039
**2010**	114	215.0	3.4e-08	196.0	2.8e-06	133.3	0.104	125.0	0.227
**2011**	33	94.1	8.6e-08	84.2	2.3e-06	55.0	0.009	37.7	0.262
**2012**	6	16.5	0.011	12.0	0.061	6.4	0.384	2.3	0.889

**Table 4:** Kruskal-Wallis statistics results for the citations case.

		*Degree*		*Betweenness*		*Closeness*		*Eigenvector*	
year	df	*χ* ^2^	*p*	*χ* ^2^	*p*	*χ* ^2^	*p*	*χ* ^2^	*p*
**1974**	7	10.3	0.173	9.5	0.221	4.3	0.750	2.8	0.907
**1975**	4	10.3	0.035	10.8	0.029	3.5	0.485	16.6	0.002
**1976**	2	2.4	0.301	3.3	0.192	0.3	0.845	1.7	0.432
**1977**	6	4.1	0.661	5.6	0.465	1.4	0.968	5.4	0.496
**1978**	3	1.2	0.749	1.6	0.661	1.2	0.755	2.6	0.453
**1979**	5	3.8	0.582	2.1	0.836	2.2	0.821	1.0	0.963
**1980**	4	1.3	0.867	2.3	0.681	5.5	0.241	3.4	0.487
**1981**	6	20.0	0.003	20.8	0.002	7.2	0.299	12.8	0.046
**1982**	3	5.9	0.115	3.9	0.269	4.4	0.218	4.3	0.233
**1983**	9	18.1	0.034	16.4	0.059	11.9	0.216	19.6	0.020
**1984**	8	11.5	0.176	12.1	0.145	7.7	0.464	19.7	0.011
**1985**	10	17.8	0.059	16.2	0.094	12.0	0.282	13.5	0.195
**1986**	9	14.0	0.121	13.5	0.142	10.4	0.323	8.5	0.484
**1987**	12	15.1	0.233	14.9	0.250	14.8	0.255	12.2	0.429
**1988**	10	34.6	1.4e-04	34.1	1.8e-04	16.1	0.097	32.0	4.0e-04
**1989**	14	34.7	0.007	32.8	0.003	18.2	0.196	28.6	0.012
**1990**	18	63.2	6.1e-07	57.1	6.0e-06	39.6	0.002	51.1	5.2e-05
**1991**	18	35.1	0.009	32.2	0.021	23.0	0.192	36.8	0.006
**1992**	21	110.8	3.4e-14	100.7	2.2e-12	81.9	3.8e-09	103.0	8.4e-13
**1993**	25	128.6	6.5e-16	118.8	3.7e-14	87.5	7.4e-09	102.0	2.8e-11
**1994**	25	140.1	5.5e-18	137.0	2.0e-17	120.3	2.0e-14	133.5	8.6e-17
**1995**	36	126.3	5.9e-12	109.6	2.3e-09	90.5	1.4e-06	139.9	3.6e-14
**1996**	38	164.2	1.2e-17	149.2	4.3e-15	106.4	2.2e-08	163.4	1.7e-17
**1997**	46	223.4	3.9e-25	211.0	5.5e-23	144.7	4.0e-12	219.6	1.8e-24
**1998**	46	159.4	2.0e-14	135.6	9.0e-11	113.3	1.3e-07	182.9	3.1e-18
**1999**	50	243.0	3.7e-27	231.6	3.5e-25	148.8	9.5e-12	221.9	1.6e-23
**2000**	66	304.2	2.8e-32	272.5	6.5e-27	179.4	2.0e-12	276.5	1.4e-27
**2001**	76	319.0	1.6e-31	266.4	5.7e-23	182.5	9.6e-11	284.3	8.2e-26
**2002**	66	364.7	6.5e-43	331.2	5.8e-37	232.2	2.3e-20	286.8	2.6e-29
**2003**	82	465.8	5.1e-55	333.7	4.4e-32	277.9	4.0e-23	342.8	1.3e-33
**2004**	94	507.3	6.0e-58	345.0	2.3e-30	269.8	6.7e-19	329.2	7.5e-28
**2005**	109	520.4	6.5e-55	398.9	1.1e-34	359.6	1.6e-28	369.7	4.4e-30
**2006**	110	489.5	2.8e-49	319.4	2.7e-22	308.1	1.1e-20	373.5	2.2e-30
**2007**	115	472.8	6.8e-45	311.8	4.5e-20	251.2	3.7e-12	296.6	5.6e-18
**2008**	111	508.5	3.5e-52	317.0	1.0e-21	254.4	2.9e-13	325.9	5.4e-23
**2009**	147	501.5	3.0e-40	316.8	1.7e-14	315.8	2.2e-14	330.4	3.9e-16
**2010**	147	382.8	6.4e-23	246.8	4.8e-07	238.3	2.8e-06	289.5	2.3e-11
**2011**	137	318.6	1.7e-16	245.8	3.4e-08	267.3	1.9e-10	245.7	3.5e-08
**2012**	33	98.7	1.8e-08	71.2	1.2e-04	70.1	1.7e-04	74.5	4.9e-05

A similar tendency is appreciated in the case of citations (Table 4). Specifically, the null hypothesis can be rejected when considering authors that start to receive citations between 1988 and 2012, with exceptions during some years, again when closeness centrality is considered. However, the interval in which we cannot deduce anything about the relation between citation patterns and centrality is extended to authors that started to receive citations between 1974 and 1988. Finally, similar conclusions are drawn when considering different measures of centrality, where there are not differences between authors who started to receive citations in one year or another (except when considering authors that started to receive citations in 1984).

### Comparisons with a null model

#### Kruskal-Wallis using a random graph as response

In order to demonstrate the significance of the results achieved, we used the Kruskal-Wallis test on a shuffled DBLP network: instead of considering the real edges in the DBLP network, we modeled these edges by means of a classic random network model, the Erdös-Rényi random graph, *G*(*n, p*) [21]. This graph, *G*(*n, p*) is defined by two parameters: the number of nodes in the graph (*n*) and the edge probability for drawing an edge between two arbitrary nodes (*p*). Our random network was obtaining using *n* = 57416 (equal to the number of authors simultaneously in both datasets) and *p* = 1.4*e*–4 (in order to have a similar level of connection than the original DBLP network), although with different distribution (random). With this test we wanted to exclude the correlation of random collaboration network distributions with our clusters. Results achieved are shown in Tables 5 and 6. According to these tables, both for the publications and citations cases, the null hypothesis cannot be rejected in almost all the the years (in which authors started to publish/receive citations) and for all the different centrality measures considered, which confirms the significance of our results.

**Table 5:** Kruskal-Wallis statistics results for the publications case (using a random graph as a response).

		*Degree*		*Betweenness*		*Closeness*		*Eigenvector*	
year	df	*χ* ^2^	*p*	*χ* ^2^	*p*	*χ* ^2^	*p*	*χ* ^2^	*p*
**1974**	7	8.2	0.312	9.6	0.212	9.5	0.222	9.6	0.214
**1975**	11	8.0	0.715	7.4	0.767	6.8	0.819	6.9	0.808
**1976**	12	6.6	0.885	6.9	0.862	9.2	0.685	9.3	0.679
**1977**	10	8.3	0.600	8.4	0.593	7.5	0.678	7.7	0.659
**1978**	14	13.7	0.474	13.3	0.505	13.6	0.479	13.3	0.502
**1979**	11	14.5	0.209	12.9	0.297	11.0	0.446	10.7	0.469
**1980**	11	7.2	0.779	8.5	0.672	9.1	0.612	9.3	0.595
**1981**	14	7.5	0.915	8.4	0.866	11.1	0.680	11.3	0.660
**1982**	16	13.1	0.663	12.5	0.707	10.8	0.821	10.8	0.823
**1983**	18	17.1	0.519	16.4	0.566	15.3	0.639	15.5	0.628
**1984**	21	21.6	0.423	22.3	0.384	24.4	0.274	24.0	0.295
**1985**	21	33.2	0.044	34.6	0.031	32.6	0.051	32.7	0.049
**1986**	21	32.4	0.054	30.5	0.082	25.5	0.224	26.0	0.207
**1987**	20	15.3	0.757	16.0	0.719	17.2	0.637	17.0	0.651
**1988**	26	23.4	0.612	22.3	0.670	22.5	0.659	22.7	0.652
**1989**	34	50.7	0.033	48.6	0.050	46.6	0.074	46.1	0.080
**1990**	25	34.1	0.106	35.9	0.073	34.5	0.098	34.4	0.099
**1991**	47	51.2	0.313	50.5	0.337	44.6	0.571	44.3	0.587
**1992**	31	33.2	0.363	32.9	0.375	30.4	0.497	30.7	0.483
**1993**	44	68.8	0.010	67.6	0.013	63.1	0.031	63.3	0.030
**1994**	51	56.1	0.289	56.4	0.280	54.9	0.329	54.6	0.338
**1995**	61	89.2	0.011	86.1	0.019	82.8	0.033	81.1	0.044
**1996**	61	76.6	0.086	75.2	0.105	76.4	0.089	76.4	0.088
**1997**	66	66.4	0.462	68.3	0.399	65.9	0.482	65.7	0.486
**1998**	78	75.7	0.551	77.8	0.485	80.2	0.410	80.1	0.414
**1999**	87	99.8	0.165	98.3	0.192	92.0	0.336	92.4	0.327
**2000**	84	96.1	0.173	97.7	0.146	97.0	0.157	96.3	0.169
**2001**	91	119.7	0.024	112.0	0.067	101.8	0.207	101.6	0.210
**2002**	107	94.2	0.807	90.8	0.869	92.2	0.846	91.4	0.860
**2003**	140	158.6	0.134	156.0	0.168	147.0	0.326	146.9	0.328
**2004**	162	165.7	0.404	161.2	0.504	158.8	0.556	159.3	0.546
**2005**	175	164.3	0.708	164.0	0.714	171.4	0.562	170.3	0.587
**2006**	179	185.7	0.350	186.0	0.344	189.5	0.282	190.3	0.268
**2007**	208	197.7	0.684	204.9	0.547	212.8	0.395	212.7	0.397
**2008**	180	240.9	0.002	241.0	0.002	231.3	0.006	232.5	0.005
**2009**	183	190.1	0.344	197.7	0.216	203.3	0.145	202.4	0.156
**2010**	114	100.6	0.810	100.3	0.817	96.1	0.886	96.2	0.885
**2011**	33	25.1	0.838	28.1	0.710	30.3	0.605	30.5	0.593
**2012**	6	5.2	0.515	4.4	0.617	3.2	0.788	3.2	0.789

**Table 6:** Kruskal-Wallis statistics results for the citations case (using a random graph as a response).

		*Degree*		*Betweenness*		*Closeness*		*Eigenvector*	
year	df	*χ* ^2^	*p*	*χ* ^2^	*p*	*χ* ^2^	*p*	*χ* ^2^	*p*
**1974**	7	8.4	0.302	8.1	0.328	8.1	0.326	8.2	0.311
**1975**	4	10.7	0.031	10.3	0.035	8.0	0.090	7.9	0.094
**1976**	2	0.5	0.767	0.4	0.817	0.2	0.883	0.3	0.868
**1977**	6	6.2	0.397	6.2	0.406	5.9	0.432	6.3	0.390
**1978**	3	2.9	0.401	3.0	0.389	3.1	0.373	3.1	0.375
**1979**	5	5.6	0.348	5.7	0.334	4.1	0.536	4.1	0.541
**1980**	4	3.5	0.473	3.6	0.464	3.3	0.505	3.6	0.470
**1981**	6	11.2	0.083	12.0	0.061	12.0	0.063	12.1	0.059
**1982**	3	1.9	0.603	2.1	0.553	2.6	0.455	2.6	0.465
**1983**	9	7.9	0.545	8.2	0.518	9.5	0.395	9.4	0.401
**1984**	8	3.7	0.881	4.1	0.851	3.6	0.891	3.7	0.885
**1985**	10	6.3	0.793	6.2	0.796	7.3	0.695	7.1	0.714
**1986**	9	4.5	0.875	5.4	0.802	7.0	0.634	7.5	0.589
**1987**	12	9.8	0.630	10.5	0.570	20.0	0.618	10.3	0.593
**1988**	10	7.6	0.671	7.1	0.712	7.5	0.680	7.4	0.686
**1989**	14	12.2	0.591	10.8	0.699	10.2	0.746	10.4	0.735
**1990**	18	24.4	0.142	24.0	0.155	21.1	0.275	20.7	0.293
**1991**	18	16.6	0.549	16.7	0.546	17.3	0.501	17.4	0.499
**1992**	21	32.8	0.049	30.3	0.086	26.8	0.177	26.1	0.202
**1993**	25	18.9	0.801	16.2	0.907	14.2	0.958	14.0	0.961
**1994**	25	26.6	0.377	25.5	0.435	23.8	0.533	24.3	0.504
**1995**	36	23.8	0.940	20.0	0.986	19.0	0.991	18.9	0.991
**1996**	38	45.2	0.197	43.0	0.265	42.8	0.271	42.8	0.273
**1997**	46	57.9	0.112	55.0	0.171	52.1	0.250	52.3	0.244
**1998**	46	36.6	0.839	36.7	0.836	36.2	0.849	36.6	0.839
**1999**	50	40.5	0.828	45.4	0.659	49.1	0.510	49.7	0.486
**2000**	66	86.7	0.045	83.3	0.074	77.2	0.163	77.0	0.167
**2001**	76	76.3	0.469	76.8	0.453	75.0	0.512	75.3	0.502
**2002**	66	85.5	0.053	84.3	0.064	84.5	0.062	84.4	0.063
**2003**	82	95.8	0.142	91.9	0.213	87.7	0.314	88.9	0.283
**2004**	94	85.2	0.731	91.2	0.562	101.3	0.286	101.9	0.271
**2005**	109	109.0	0.482	106.3	0.556	106.5	0.550	106.3	0.555
**2006**	110	107.9	0.539	109.4	0.497	114.7	0.360	115.1	0.352
**2007**	115	148.2	0.020	156.5	0.006	154.3	0.008	154.1	0.009
**2008**	111	129.9	0.106	130.1	0.104	129.8	0.104	128.6	0.121
**2009**	147	139.6	0.656	140.0	0.646	140.2	0.641	139.8	0.651
**2010**	147	169.1	0.102	166.5	0.129	161.0	0.203	162.2	0.185
**2011**	137	161.6	0.074	162.5	0.067	163.4	0.061	164.5	0.055
**2012**	33	30.4	0.598	32.1	0.514	37.2	0.283	36.8	0.298

#### Correlation measures between authors’ centrality and their publications (citations) counts

**Fig 2:**
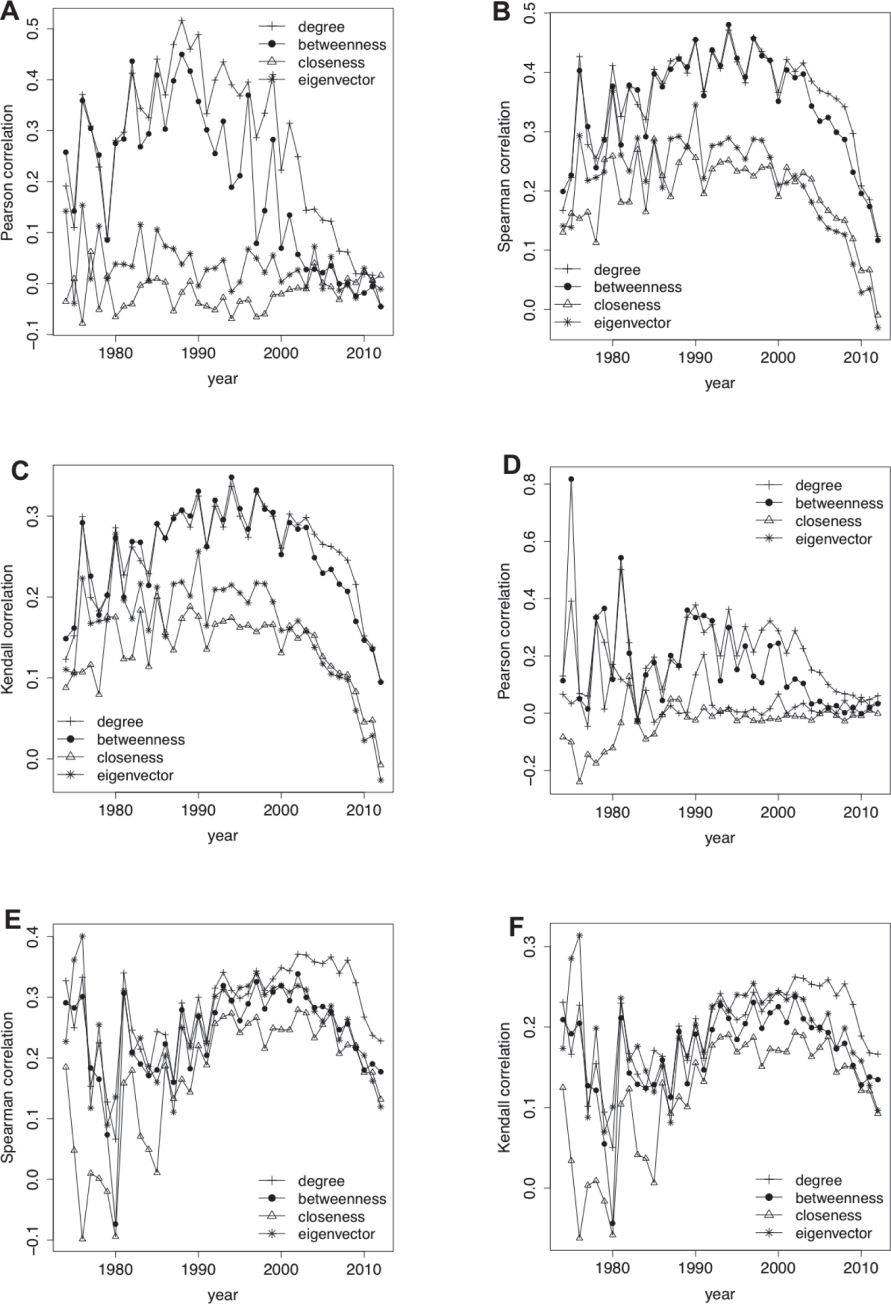
Correlation measures between authors’ centrality and their publications (citations) count. (a) Pearson correlation between authors’ centrality and their publications count. (b) Spearman correlation between authors’ centrality and their publications count. (c) Kendall correlation between authors’ centrality and their publications count. (d) Pearson correlation between authors’ centrality and their citations count. (e) Spearman correlation between authors’ centrality and their citations count. (f) Kendall correlation between authors’ centrality and their citations count.

With the aim of testing the relation between authors’ centrality in the co-authors network and their total number of publications (citations), we calculated three well-known correlation indexes: Pearson, Spearman and Kendall. Fig. 2 shows the values of the different indexes taking into account different centrality measures (degree, betweenness, closeness and eigenvector centrality) and splitting authors according to the year of their first publication/citation. Results revealed that the correlation between centrality and number of publications (citations) is not significative (lower than 0.5 in almost cases), with the only exception of the authors who started their activity around 1990 when considering the correlation between their publications and degree (betweenness) centrality (Fig. 2A and Fig. 2B). This justifies the necessity of considering the whole scholars’ timeline as sign of their research activity.

**Other experiments without considering time**. Finally, we run an experiment to demonstrate the importance of classifying authors according to the year of their first publications/citation when looking for their relation with centrality in the co-authorship network. Specifically, we computed the Pearson, Spearman and Kendall correlation indexes between the number of publications/citations and the degree centrality (the one with best results in Fig. 2) of all the authors in the dataset (independently of the length of their careers). Results in Table 7, with correlation values within the ranges of the ones in Fig. 2 confirm the importance of splitting authors by the year in which their careers started.

**Table 7:** Correlation measures between authors’ centrality and their publications (citations) counts without splitting authors per year of their first publication (citation).

	**Publications**	**Citations**
**Pearson**	0.2420822	0.1973301
**Spearman**	0.4146143	0.3929034
**Kendall**	0.303451	0.2848471

## Discussion

The main focus of this work is on analysing the importance of collaborations in academic success over time, by considering success as the volume of publications and cites to these publications. That is, on analysing if the volume of collaborations of one author together with the relevance of his collaborators is related to his research performance over time. We made use of two different datasets: one obtained by crawling the *Google Scholar* platform and the other the well-known DBLP dataset. By the application of a two-steps methodology based on (i) clustering scholars’ timelines to explore their temporal patterns and (ii) the application of non-parametric statistics to establish the correlation among timeline and centrality, we confirmed our hypothesis that computer scientists’ centrality in the co-authorship network is related with the patterns that their publications (citations) followed along the years. Although this relation was found both in the case of publications and citations and independently of the centrality measure, we could not guarantee the existence of relation (i) for scholars that started to publish/receive citations recently (very short time series) neither (ii) for those that started to publish before 1979 in the case of publications (1988 in the case of citations). This relation holds for most of the period considered: this confirms our initial hypothesis that collaboration affects (and is affected by) research performance over time. What is likely is that, the more collaborations one author has, the higher his number of publications will be and the more attention they will receive. Moreover, the relevance of these collaborations seems to play a key role in the increase the number of publications and their visibility.

With respect to future work, our analysis could be extended in three different ways. Firstly, our mechanism for matching authors in the datasets could be improved by (i) taking into account measures of lexicographic distance between strings as the Levenshtein distance and (ii) applying techniques to detect ambiguous author names in scholarly data as the one proposed by Sun et al. [22, 23]. At the moment authors with the same name are considered the same person while by using the aforementioned techniques, we expect to increase the number of authors simultaneously in both datasets and, therefore, increasing the size of the sample, besides avoiding false positives in the matching-mechanism. Secondly, by considering other network measures, such as cross-clustering coefficient and cross-transitivity [24, 25] to conduct social network analysis to see whether more further information could be uncovered. Finally, once temporal series are classified in clusters, it could be interesting to characterise those series according to the different types of continuous dynamics that they exhibit: periodic, chaotic, and periodic with noisy [26–28].

To conclude, the extension of our study to other disciplines besides Computer Science would give us a better understanding of the dynamics of research. Once discovered these dynamics, the next logical step should be the development of a prediction framework that allows scholars to improve their collaboration network in order to increase their temporal success.

## Supporting Information

S1 Appendix Anatomy of the Google Scholar dataset. (PDF)
