# Supplementary material for: The Evolution of Your Success Lies at the Centre of Your Co-Authorship Network
Source: PLoS One. 2015 Mar 11;10(3):e0114302. doi: 10.1371/journal.pone.0114302 (PMC4356565; doi:10.1371/journal.pone.0114302)
Supplement: S1 Appendix — (PDF) [file pone.0114302.s001.pdf]

Appendix S1: Anatomy of the Google Scholar dataset

In order to provide a complete description of our Google Scholar dataset, we run a batch of well-known analysis over it and present the summary. Please, note that in the analysis results showed below we distinguish between (i) authors in the entire dataset -all disciplines- and (ii) authors simultaneously in the dataset and in DBLP -computer science-. The analysis results of the DBLP dataset were avoided since they are extensively provided in previous works [1–3].

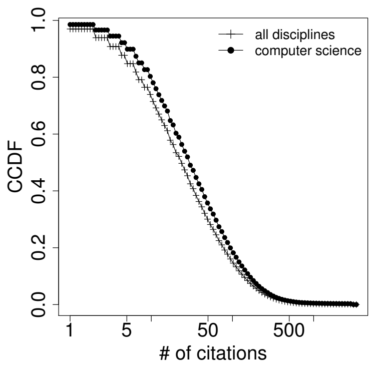

(a) # of publications per author (considering authors working on any scientific discipline -*all disciplines*- and only *Computer Science* authors).

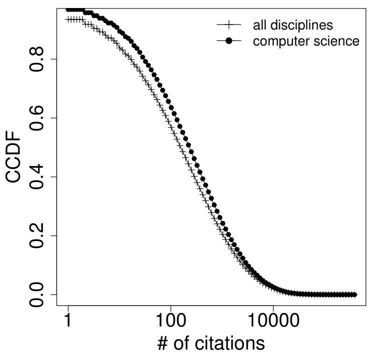

(b) # of citations per author (considering authors working on any scientific discipline -*all disciplines*- and only *Computer Science* authors).

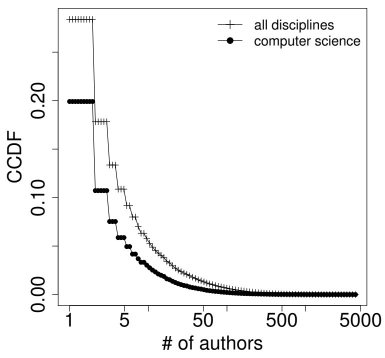

(c) # of authors per area of interest (considering authors working on any scientific discipline -*all disciplines*- and only *Computer Science* authors).

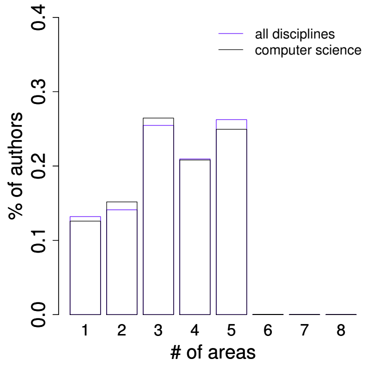

(d) # of areas of interest per author (considering authors working on any scientific discipline -*all disciplines*- and only Computer Science authors).

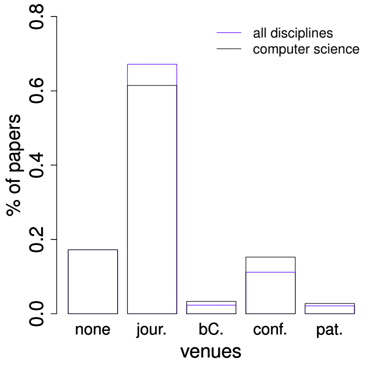

(e) # of publications per venue (considering authors working on any scientific discipline -*all disciplines*- and only Computer Science authors).

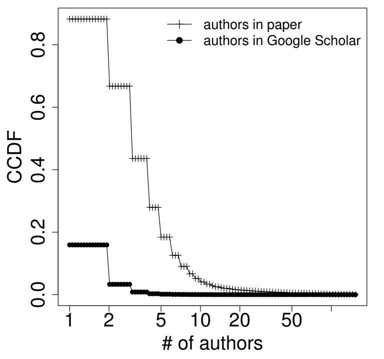

(f) # of authors per publication (considering authors working on any scientific discipline -*all disciplines*- and only Computer Science authors).

Figure 1. Anatomy of the Google Scholar dataset.

Among all the information available in Google Scholar, the **authors' publications and the cita-**

tions to these publications are the most relevant for our interests. Figures 1a and 1b contain some numerical data about this information. Specifically, Figure 1a contains the distribution of the number of publications of authors, meanwhile Figure 1b displays the distribution of the citations that these authors have received (both in logarithmic scale). With respect to publications, the 70% of the authors in the entire dataset have less than 50 publications, being this percentage scarcely lower in the case of computer science authors. Also, the mean of publications per author in computer science (73.68) is slightly higher than the mean of publications per author in the entire dataset (58.73). With regard to citations, there is relatively large difference between computer science authors and all the authors in the entire dataset, since in the entire dataset around half of the authors have less than 150 citations while in the case of computer science authors this number increases until 225 citations. Talking in terms of means, the publications of an author in the entire dataset have received, in mean, 1130 citations, whereas the ones of a computer science author have received around 1337.

Areas of interest in Google Scholar can be useful to locate experts in a given domain since, when searching for authors interested in a given area, Google Scholar displays them in decreasing order of citations received. But the indication of areas of interest in Google Scholar is up to authors, and not all authors decide to identify them. On the same way, the number of areas and their names are not prefixed and so, again, each author is free to choose them. But, with all of these, there are areas shared by several authors. Figure 1c displays the CCDF of **number of authors per area of interest** using a logarithmic scale. In this case, as the x axis follows a logarithmic scale, the maximum of the CCDF function (which occurs in $x = 0$) is not represented. Also, this figure reveals that, as authors are free when indicating their areas, most of them state very specific areas, which makes that most of the areas obtained only include one author. Therefore, the percentage of areas with more than one author is around 20% in the entire dataset, whereas, in the case of computer science areas, this percentage increases until being close to 30%.

In the same line, Figure 1d shows the distribution of the **number of areas of interest indicated per author**. As we see, most authors indicated between 3 and 5 areas of interest. Since for obtaining the Google Scholar dataset we discovered the new authors to crawl through their areas of interest, all the authors in the dataset have indicated, at least, one area.

Finally, publications in Google Scholar can be classified as articles in conferences (*conf.*), journals (*jour.*), patents (*pat.*) and book chapters (*bC.*). Figure 1e displays the distribution of **publications attending to their venues**. Please, note that *none* means that the publication has not been classified into any venue. Results show a clear superiority of journals in terms of coverage, followed at large distance by conferences. As expected, the difference between the journal publications with respect to the conference publications is lower in the case of computer science than in the entire dataset.

Each publication in our dataset has, at least, one author with profile in Google Scholar. But it does not guarantee that all the authors of these publications have a profile in this platform. The difference between the distributions of (i) the **number of authors in a publication and (ii) the number of authors in a publication that have a profile in Google Scholar** gives us an idea of the coverage of Google Scholar. Figure 1f displays both distributions for the case of computer scientists. When considering the number of authors per publication, the percentage of publications with 3 or more authors is higher than 40% whereas, in the case of considering only authors with a profile in Google Scholar, this percentage decreases until being close to 0. As the connections in the co-authorship network take place when two authors (nodes in the network) have co-authorised, at least, one publication, the fact that not knowing all their shared publications hampers us from having the real well-connected network. This, together with the absence of all the nodes in the co-authorship network (not all the scholars have a profile in Google Scholar), have implied the necessity of an alternative dataset for extracting the co-authorship network which, in our case, is the well-known DBLP dataset.

Finally, Figure 2 shows the number of co-authors per author, i.e. the degree centrality of the nodes in the network of co-authors. Specifically, it shows the number of co-authors (i) manually indicated by

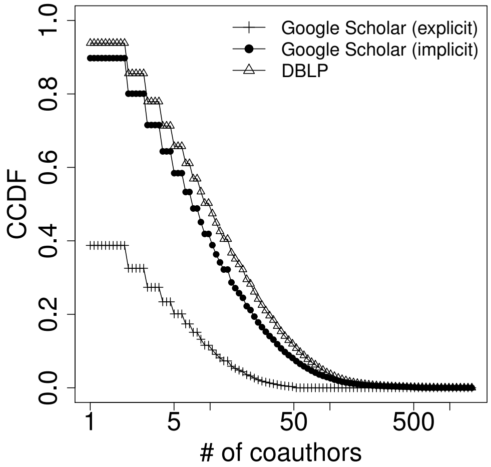

Figure 2. Number of coauthors per author (considering co-authors (i) indicated in Google Scholar profiles - *explicit*- (ii) obtained from common publications in Google Scholar -*implicit*- and obtained from common publications in *DBLP*).

authors in their Google Scholar profiles (explicit), (ii) extracted from common publications in Google Scholar (implicit) and (iii) extracted from common publications in DBLP. As the use of the DBLP datasets limits our study to computer science authors, henceforth we will use and show results over data from authors who are simultaneously in both datasets (Google Scholar and DBLP). Focusing on the figure, the difference between the distribution of the centrality degree in the "explicit" case with respect to the other two distributions indicates that, as aforementioned, authors are not keen on indicating their co-authors in their profiles. So, the co-authorship network inferred by considering the manually included co-authors in Google Scholar is not complete and its use for calculating scholars' location would let us to erroneous results. Finally, the distribution curves in the case of considering common publications in Google Scholar (implicit) and in DBLP exhibits a similar tendency, although the average degree centrality is lower in the case of Google Scholar (18.36 compared to the 23.03 in DBLP).

References

1. Martin T, Ball B, Karrer B, Newman M (2013) Coauthorship and citation in scientific publishing. CoRR abs/1304.0473.

2. Ley M (2009) DBLP: some lessons learned. Proceedings of the VLDB Endowment 2: 1493–1500.

3. Reitz F, Hoffmann O (2010) An analysis of the evolving coverage of computer science sub-fields in the DBLP digital library. In: Research and Advanced Technology for Digital Libraries, Springer. pp. 216–227.
